# Effects of co-infection on the clinical outcomes of *Clostridium difficile* infection

**DOI:** 10.1186/s13099-020-00348-7

**Published:** 2020-02-25

**Authors:** Muhammad Shafiq, Hani Alturkmani, Yousaf Zafar, Vishal Mittal, Hafsa Lodhi, Waqas Ullah, Joseph Brewer

**Affiliations:** 1 University of Kansas Medical Center, 4000 Cambridge St, 6040 Delp, MS 1020, Kansas City, KS 66160 USA; 2 Truman Medical Center, 2301 Holmes Street, Kansas City, MO 64108 USA; 3 NCH Healthcare System, 311 9th Street North, Suite 201, Naples, FL 34102 USA; 4 University of Missouri Kansas City, 2411 Holmes Street, Kansas City, MO 64108 USA; 5 St. Louis Children’s Hospital, One Children’s Place, CB 8116, St. Louis, MO 63110 USA; 6 Abington Hospital-Jefferson Health, 1200 Old York Road, Abington, PA 19001 USA; 7 Saint Luke’s Hospital, 4401 Wornall Rd, Kansas City, MO 64111 USA

**Keywords:** *Clostridium difficile*, Co-infection, Clinical outcomes

## Abstract

**Background:**

*Clostridium difficile* (*C. difficile*) is a spore-forming, Gram-positive rod that is known to be associated with antibiotic use. It is one of the leading causes of nosocomial diarrhea in the industrialized world and therefore warrants further study of its nature. It isn’t clear if co-infection by other organisms can affect the outcome of *C. difficile* infection (CDI).

**Methods:**

A single center retrospective study was done and it used inclusion criteria of 18 years of age and being tested positive for CDI on FilmArray® multiplex gastro-intestinal (GI) panel. Exclusion criteria were a GI panel performed on an outpatient basis, recurrent CDI, and the presence of end-stage renal disease, cirrhosis, or a non-GI infection. The stool sample for all patients were collected within 48 h of presentation to the hospital. There were 235 of 2576 GI panels selected for a retrospective chart review based on the above criteria. Among these 235 patients, 38 had a co-infection (CDI+ another GI infection = group A or cases) and the rest had only CDI (group B or controls). Group A was compared with group B for CDI severity, its response to treatment, recurrence, and length of the hospital stay, using 0.05 as the alpha criterion.

**Results:**

Most patients with CDI were female and above the age of 60 years. Co infection did not increase the severity of CDI based both on the American College of Gastroenterology criteria (p 0.16) as well as Infectious Disease Society of America criteria (p 0.77). Co infection group also didn’t have significantly different CDI related treatment failure rate (p 0.23), or CDI recurrence rate (p 0.49). Co-infection was also not associated with lengthier hospital stay (p 0.41).

**Conclusion:**

Our study suggests that co-infection doesn’t affect the severity of CDI or can cause treatment failures. Additionally, there was no significant increase in hospital stay, or increase in CDI recurrence associated with co-infection. Therefore, if CDI is the leading clinical diagnosis and a patient is tested positive for co-infection in addition to CDI on FilmArray® multiplex GI panel, this co-infection shouldn’t change the management for CDI. Limitations of this study (including retrospective nature of the study, small sample size, single site study, not including all microbiome and non-inclusion of race) should also be taken into account, while considering the applicability of the results of this study.

## Background

*Clostridium difficile* infection (CDI) poses a major burden on the patient and the healthcare system by increasing mortality, morbidity, length of hospital stay, and costs [1–3]. There has been a steady increase in the incidence of CDI since 2000, with an associated increase in severity and poor clinical outcomes [4]. A European study showed that one in ten patients with CDI were either transferred to the intensive care unit, underwent a colectomy, or died as a result of the infection [5]. This requires further understanding of the pathophysiological and clinical aspects of CDI, which might help physicians to provide better care for patients with CDI, and it may also reduce the burden on the health care system.

## Methods

A single center retrospective study was conducted at Saint Luke’s Hospital of Kansas City, MO—USA. Among the 2576 FilmArray® multiplex gastrointestinal (GI) panels performed from January 1, 2015 to December 31, 2016, 235 patients were selected for chart review based on the inclusion and exclusion criteria. Inclusion criteria included age of 18 years or above who tested positive for *Clostridium difficile* (*C. difficile*) via the FilmArray® multiplex GI panel. Exclusion criteria included GI panel performed on an outpatient basis; recurrent CDI; and patients with end-stage renal disease (ESRD), cirrhosis or non-GI infection.

Selected patients’ population was divided into two groups. Patients who had CDI as well as another GI infection (co-infection) were placed into the case group (group A). Patients who had CDI only were placed into the control group (group B). Cases (co-infections or group A) were then compared with controls (CDI only or group B) to investigate the association between co-infection and CDI severity, its response to treatment, CDI recurrence, and length of hospital stay. The Chi-square test and Fisher’s exact tests were used to identify any statistical significance for severity, response to treatment, and CDI recurrence, with an alpha criterion of 0.05. An independent *t*-test was used to assess for any statistical significance in the duration of hospital stay, with 0.05 as the alpha criterion.

## Results

Among the 235 patients selected based on the inclusion and exclusion criteria listed above, 93 patients (39.57%) were male and 142 patients (60.43%) were female. Two patients (0.85%) were younger than 20 years of age, 13 patients (5.53%) were 20–29 years old, 24 patients (10.21%) were 30–39 years old, 19 patients (8.09%) were 40–49 years old, 26 patients (11.06%) were 50–59 years old, 47 patients (20%) were 60–69 years old, and 104 patients (44.26%) were 70 years old or above.

Among 235 patients, there were 38 cases (16.17%) and 197 controls (83.83%). Among the 38 cases (patients with co-infection or group A), two patients had three infections at one time (*C. difficile* plus two other organisms). The rest had only one more infectious agent besides *C. difficile*.

The frequency of co-infection in cases was variable. Enteropathogenic *Escherichia coli *(*E. coli*)* was* the source of co-infection in 14 patients, norovirus in nine patients, *Campylobacter jejuni* in five patients and enterotoxigenic *E. coli* in three patients. Enteroaggregative *E. coli, Salmonella* and sapovirus were source of co-infection in two patients each while enteroinvasive *E. coli, Yersinia enterocolitica and* adenovirus in one patient each.

**Fig. 1:**
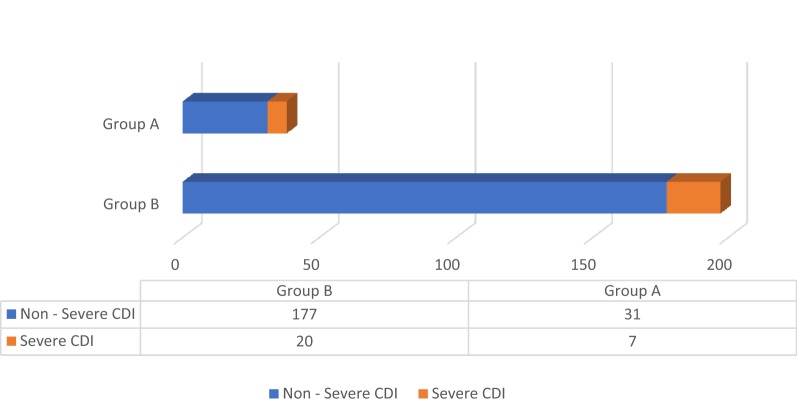
Severity based on the criteria of American College of Gastroenterology (*p*-value = 0.16)

**Fig. 2:**
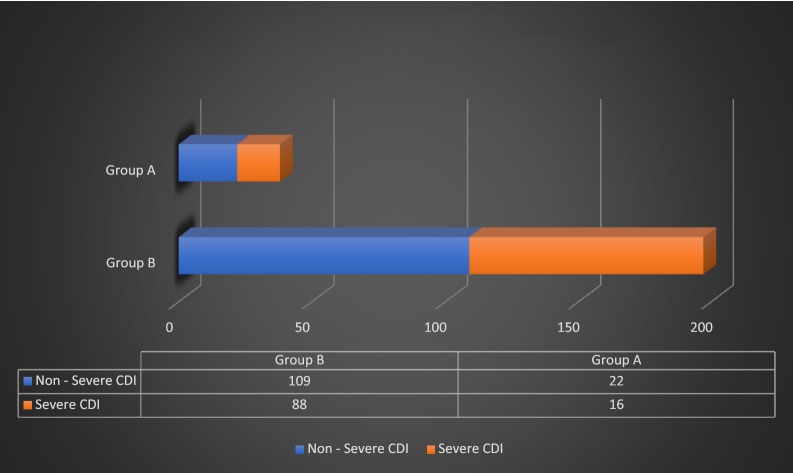
Severity based on the criteria of Infectious Disease Society of America (*p*-value = 0.77)

**Fig. 3:**
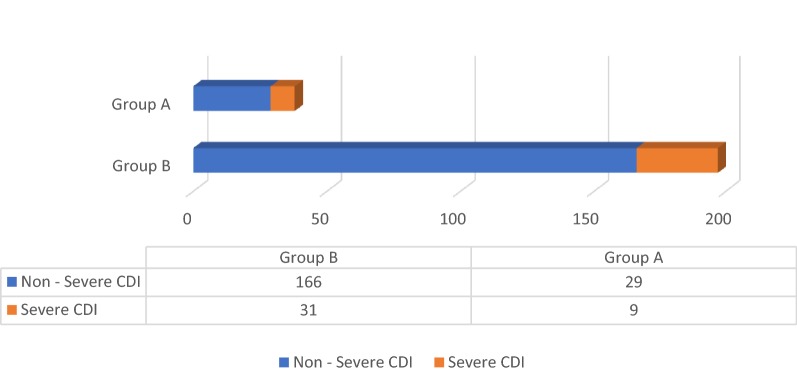
Treatment escalation (*p*-value = 0.23)

There was no statistically significant difference in the severity of CDI between the two groups based on the American College of Gastroenterology (ACG) criteria (p 0.16) as well as Infectious Disease Society of America (IDSA) criteria (p 0.77) as shown in Figs. 1 and 2. There was no significant difference in treatment failure/escalation either between the two groups (p 0.23) as shown in Fig. 3.

After enteropathogenic *E. coli*, norovirus was the most common co-infection in this study. Its sub-set analysis showed that among the nine patients with norovirus co-infection, six were male and three were women. Only two patients were below 60 years of age. One patient with norovirus co-infection had severe infection based on both IDSA and ACG criteria while three other patient had severe infection based on IDSA criteria only. Only one out of nine patients required escalation of treatment. None of these nine patients had recurrence of CDI. Among these patients, average duration of hospital stay was 4.88 days, not significantly different than for the whole group A (cases).

**Fig. 4:**
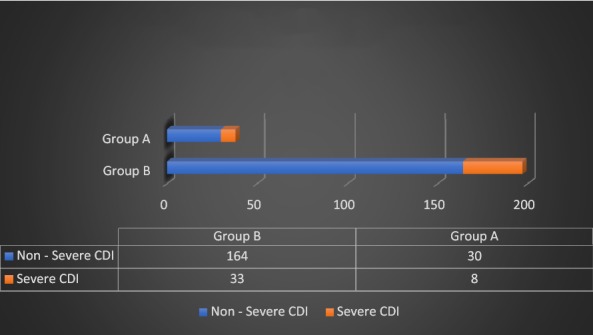
*Clostridium difficile* infection recurrence (*p*-value = 0.49)

**Fig. 5:**
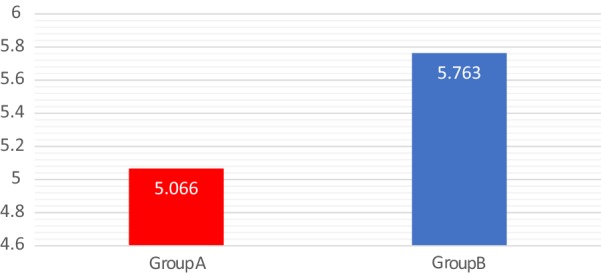
Mean duration of hospital stay in days (*p*-value = 0.41)

Likewise, there was no significant difference in *C. difficile* recurrence rate between the two groups (p 0.49) as detailed in Fig. 4. The mean hospital stay for the patients in case and control groups was comparable with no significant difference (p 0.41) between the two groups as show in Fig. 5.

## Discussion

Many studies have investigated the factors that predispose patients to CDI^3^ and its recurrence after the initial treatment [6]. Some recent studies have also investigated the co-infection rate with CDI [7–9]. According to a multicenter evaluation of the BioFire FilmArray® GI panel for etiologic diagnosis of infectious gastroenteritis [9], at least one potential pathogen was detected in 53.5% of the stool specimens that were collected, and among the positive samples, 31.5% tested positive for more than one potential pathogen. The samples that were co-infected showed that CDI was present in 53.4% of them. Some studies on pediatric population reported that CDI and co-infection are associated with increased severity [10]. However, only a few studies on adult populations have assessed the effects of co-infection on the severity of CDI [10, 11] and other clinical outcomes [12].

Our study aimed to determine the burden and effects of co-infection on CDI including CDI severity, its response to treatment, CDI recurrence, and length of the hospital stay in adult population.

We followed both ACG as well as IDSA criteria to define the severity of CDI. This included a serum albumin < 3.0 g/dL plus either a white blood cell (WBC) count ≥ 15,000 cells/mm^3^ or abdominal tenderness for ACG severity criteria. Severity of CDI based on the IDSA criteria was leukocytosis with a WBC count > 15,000 cells/mm^3^ or a serum creatinine level ≥ 1.5 times the premorbid level. Failure to respond to initial treatment was determined as any step up in the treatment regimen. For example; if a patient was started on intravenous or oral metronidazole with no improvement of symptoms and therefore was switched to oral vancomycin, was considered to be a failure of the initial treatment. Similarly, patients requiring increase of vancomycin dose or switch of oral vancomycin to oral fidaxomicin was considered to be treatment failure or treatment escalation. Recurrence of CDI was defined as reappearance of symptoms and being tested positive for CDI after a complete recovery of diarrhea for at least two weeks. Duration of hospital stay was measured in days for all patients and was compared in both groups.

The idea behind using both the ACG and IDSA criteria to define severity was to allow the results to be interpreted in a more universal or generalized manner. We also minimized the confounding variables as much as possible. For example, in cirrhosis, albumin levels are low and this affects the interpretation of severity based on the ACG criteria. Thus, these patients were excluded. Similarly, creatinine in patients with ESRD has less meaning, but creatinine is part of the IDSA criteria for severity. Thus, these patients were also excluded. Patients with a concomitant non-GI infection were also excluded because they cause an independent increase in the WBC count and the WBC count is part of the severity criteria for both ACG and IDSA. Recurrent CDI is more challenging to treat than the initial CDI [13]. Therefore, if a patient with a recurrent CDI was to be compared to a patient with an initial CDI, the treatment for the recurrent CDI would be more likely to fail and these patients are expected to have a longer duration of hospital stay, regardless of the presence of a co-infection, compared with patients with an initial infection. To eliminate this bias/confounding factor, all patients with recurrent CDI were excluded and only patients with an initial CDI were included.

Surprisingly, in contrast to the previous studies, our results showed that co-infection in adult population does not affect outcomes in terms of CDI severity, its response to treatment, CDI recurrence, and length of hospital stay. Colonization with certain pathogenic bacteria, such as *E. coli*, has been demonstrated in literature at this point [14]. No objective data is available but it is a possibility that the co-infections in our study represent colonization or asymptomatic infections and they were detected because of the higher sensitivity of polymerase chain reactions employed by the FilmArray® multiplex GI panel [9]. This information can help to prevent unnecessary treatment escalation in CDI patients if another co-infection is detected via FilmArray® multiplex GI panel, especially if CDI is the leading clinical diagnosis. Additionally, if such patient’s CDI worsens, it is less likely to be the result of co-infection and an alternative cause should be investigated.

## Conclusion

If CDI is the leading clinical diagnosis and a patient is tested positive for co-infection in addition to CDI on FilmArray® multiplex GI panel, this co-infection shouldn’t change the management for CDI. Given additional limitations of this study as outlined below, careful consideration has to be given and further studies must be taken in to account for sick patients (such as patients in intensive care units), patients with co-infection who are not improving with treatment for CDI alone and patients with additional co-morbidities (such as patients with ESRD, cirrhosis, inflammatory bowel disease, patients on chronic immune-suppressions or patients with human immunodeficiency virus).

## Limitations

Although efforts were made to minimize confounding variables and evaluate the effects of co-infection on the outcomes of CDI, there are still some limitations to this study. This was a retrospective chart review study. So, randomization could not be done and only reported outcomes were recorded. Due to our strict selection criteria, the sample size was reduced to 38 patients having co-infection (cases) and therefore it didn’t include all microbiome. Race as demographic parameter wasn’t included. Events only reported to our institute were recorded as it was a single center study posing a risk of reporting bias.

## Data Availability

All data generated or analyzed during this study are included in this published article.

## Abbreviations

ACG: American College of Gastroenterology
*C. difficile*: *Clostridium difficile*
CDI: *Clostridium difficile* infection
*E. coli*: *Escherichia coli*
ESRD: End-stage renal disease
GI: Gastro-intestinal
IDSA: Infectious Disease Society of America
WBC: White blood cell
